# 创伤后获得性血友病A：1例报告并文献复习

**DOI:** 10.3760/cma.j.cn121090-20241223-00581

**Published:** 2024-12

**Authors:** 燕燕 谢, 美荣 杨, 明珠 张, 昭玲 邓, 霖虹 王, 金玉 李, 俊暕 赵, 宝来 华, 振宇 闫

**Affiliations:** 1 华北理工大学附属医院血液科，唐山 063000 Department of Hematology, North China University of Science and Technology Affiliated Hospital, Tangshan 063000, China; 2 华北理工大学附属医院烧伤科，唐山 063000 Department of Burn, Affiliated Hospital of North China University of Science and Technology, Tangshan 063000, China; 3 华北理工大学附属医院重症医学科，唐山 063000 Intensive Care Unit, North China University of Science and Technology Affiliated Hospital, Tangshan 063000, China; 4 华北理工大学附属医院检验科，唐山 063000 Department of Laboratory Medicine, Affiliated Hospital of North China University of Science and Technology Tangshan 063000, China; 5 北京世纪坛医院血液内科，北京 100038 Department of Hematology, BeiJing ShiJiTan Hospital, Beijing 100038, China

## Abstract

获得性血友病A（AHA）是一种由于循环血中出现抗凝血因子Ⅷ（FⅧ）自身抗体导致FⅧ活性（FⅧ∶C）降低的获得性出血性疾病。本文报道1例创伤后AHA的患者，经去除抗体及旁路替代治疗后快速缓解，外科干预后伤口愈合，并进行相关文献复习，以期提高临床医师对于创伤后AHA的认识，做到及早诊断及早治疗。

## Abstract (en)

Acquired Hemophilia A is an acquired bleeding disorder characterized by reduced FⅧ activity due to the presence of autoantibodies against anticoagulant factor Ⅷ in the circulation. Laboratory tests are typically characterized by prolonged isolated activated partial thromboplastin time（APTT）. Clinically, it often manifests as severe bleeding, and 50％ of AHA patients can identify the cause. This article reports on a patient with post-traumatic AHA who rapidly improved after antibody removal and bypass replacement therapy, followed by wound healing following surgical intervention. A review of relevant literature is also conducted to enhance clinicians' awareness of AHA, which presents with normal coagulation initially and gradually develops into prolonged APTT accompanied by bleeding manifestations, aiming for early diagnosis and timely treatment.

获得性血友病A（AHA）是一种由于循环血中出现抗凝血因子Ⅷ（FⅧ）自身抗体导致FⅧ活性（FⅧ∶C）降低的获得性出血性疾病。其特点为既往无出血史和无阳性家族史的患者出现自发性出血或者在手术、外伤或侵入性检查时发生异常出血。实验室检查以孤立性活化的部分凝血活酶时间（APTT）延长为特征。大约有50％的AHA患者可以发现病因或基础疾病，如自身免疫性疾病、恶性肿瘤、药物、感染、妊娠以及创伤等，AHA治疗成功的关键在于及时诊断、及早给予恰当的治疗。本文报道1例创伤后AHA的患者，经去除抗体及旁路替代治疗后快速缓解，外科干预后伤口愈合，并进行相关文献复习，以期提高临床医师对于前期凝血正常，逐渐出现APTT延长合并出血表现的AHA的认识，做到及早诊断及早治疗，同时提示外科医师未在替代治疗支持下禁忌有创操作，避免发生危及生命大出血。

## 病例资料

患者，男，69岁。2024年2月29日因车祸致头外伤，伤后意识不清，无呕吐，入外院急诊科查体：血压90/60 mmHg，意识模糊，刺激有逃避反应，右眼瞳孔直径4 mm，左眼3 mm，直接对光反射正常，间接光反射不能查，骨盆区肿胀，右小腿可见开放性伤口，有陈旧性血性溢出，双小腿畸形，可及骨擦感。CT：蛛网膜下腔出血，右侧额叶脑挫裂伤，顶部头皮软组织肿胀，额骨骨折，眼眶多发骨折，胸4椎体骨折，右侧耻骨下支骨折，右肺中叶、两肺下叶炎症。X线片示双侧胫腓骨骨折。急诊眼科会诊无特殊处理，急诊创伤科予以缝合下肢伤口，给予止血及补液治疗，肌肉注射破伤风抗毒素后收住外院神经外科病房。既往体健，2年前行前列腺增生手术。否认肝炎史、伤寒史、结核史，否认高血压史、糖尿病史、脑血管病史、精血史神病史，否认输血史，否认过敏史。

入院后血常规：WBC 23.15×10^9^/L，HGB 98 g/L，PLT 169×10^9^/L。凝血系列：PT 14.2s，APTT 37.4 s，Fib 4.27 g/L。给予气管切开、去甲肾上腺素维持生命体征，蛇毒血凝酶止血多发伤采取保守治疗。入院后患者发热，胸部CT提示肺炎，给予头孢呋辛抗感染，效果不佳，痰培养提示鲍曼不动杆菌、肺炎克雷伯菌、粘滞沙雷菌，先后调整为哌拉西林/他唑巴坦、头孢哌酮舒巴坦、去甲万古霉素、亚胺培南、利奈唑胺、莫西沙星，体温逐渐控制，肺炎逐渐吸收。住院期间监测血常规示HGB 83～98 g/L，APTT持续在正常范围。3月15日患者突发腹痛，腹部CT提示脾破裂，复查血常规HGB降至50 g/L，APTT正常，紧急行剖腹探查术+全脾切除术，术中输红细胞3 U、血浆680 ml，术后无异常出血，复查HGB逐渐升至93 g/L。病情稳定后行择期双侧胫骨骨折切开复位钢板内固定术+右胫骨平台骨折切开复位钢板内固定术+右髌骨撕脱骨折切开复位内固定术，术中出血400 ml，输血4.5 U、血浆600 ml。3月22日复查HGB 105 g/L，APTT 35.9 s。3月24日APTT升至65.9 s，3-31 APTT升至59.1 s，HGB 90 g/L左右。

2024年4月2日患者右大腿局部肿胀，进行性加重。查体：皮温高，压痛，边界清楚，当天行肌肉超声提示血肿：直径长达23.3 cm×6.58 cm。4月3日复查HGB降至62 g/L，APTT 88.7 s，当时抗生素应用头孢哌酮/舒巴坦抗感染，发现APTT延长后调整为哌拉西林/他唑巴坦，同时给予氨甲环酸、维生素K1、尖吻蝮蛇血凝酶、输血、血浆、冷沉淀支持，输血浆及冷沉淀支持下当天复查APTT缩短3 s～10 s，次日复查再次明显延长，HGB进行性下降，4月6日APTT 96 s，患者转院至北京某医院急诊科，给予营养支持、利奈唑胺0.6 g每12 h 1次抗感染治疗，右下肢血管造影未发现明确出血点，凝血因子活性检查当时未回报，因重症医学科无床位，患者于4月7日再次回当地医院继续治疗。右大腿肿胀，肿胀范围上至右髋关节下10 cm，下至右膝关节上10 cm，前外侧张力大，左大腿及双小腿无明显肿胀，返院当天APTT升至115.7 s，HGB 74 g/L，4月8日行右大腿血肿切开减压手术，术中未见出血点，肌肉持续渗血不止，血压降至80/50 mmHg，多巴胺维持血压，每天输注血浆800～1000 ml、冷沉淀6～10 U、红细胞2～6 U，每天切口辅料全部浸透，HGB最低降至48 g/L，APTT 88 s左右，4月11日FⅧ∶C 3.2％，在输注血浆、冷沉淀基础上联合应用血源性FⅧ 1000 U，仍不能有效止血，APTT仍显著延长。

2024年4月16日由当地医院转入我院重症医学科，入院后生命体征稳定，带入气管切开套管、胆囊造瘘管，右下肢大腿纱布辅料见血性渗出伴异味。入院诊断：凝血异常待查，AHA可能性大；右大腿血肿清除减张+清创缝合术后；肺炎；多发肋骨骨折；肺挫伤；创伤性蛛网膜下腔出血；硬膜下血肿；脑挫裂伤；骨盆骨折；右侧耻骨骨折；左侧髋臼骨折；胸3、4、6、10椎体压缩性骨折；右侧旋股外侧动脉横支动脉瘤；弥漫轴索损伤；额骨骨折；前颅窝底部骨折；右眼眶上壁内侧壁骨折；右侧上领窦额突骨折；下肢深静脉血栓形成；两侧胸腔积液；双膝关节囊积液；胆囊结石；气管切开术后；胆囊造瘘术后；脾切除术后；双侧胫骨右侧胫骨平台右髌骨骨折切开复位内固定术后。入院后查血常规：WBC 20.1×10^9^/L，HGB 72 g/L，PLT 222×10^9^/L。凝血系列：PT 16.2 s，APTT 79.5 s，Fib 4.27 g/L，D-二聚体9 326 µg/L。APTT 1∶1纠正试验，即刻纠正53.2 s，温浴2 h后99.9 s。狼疮抗凝物、抗磷脂抗体阴性。下肢静脉血管超声：双下肢小腿肌间静脉血栓。复查FⅧ∶C 24.2％，FⅧ抑制物0.97 Bethesda。诊断：AHA。4月16日输注红细胞2 U，4月17日请烧伤科协助术口清创换药，取出填塞纱布，创口达20 cm，可见活动性出血，伴少量血凝块，异味明显，请血液科会诊后给予重组活化凝血因子Ⅶ（rFⅦa）5 mg静脉注射、凝血酶原复合物（PCC）2400 U/d、甲泼尼龙40 mg/d、环磷酰胺片100 mg/d口服。4月18日突发寒战，考虑血流感染，行血培养，给予头孢他啶阿维巴坦2.5 g静点每8 h 1次抗感染治疗，换药过程中仍有活动性出血，APTT 64.5 s，D-二聚体8 058 µg/L，将PCC加量至3 600 U每日1次，HGB最低降至57 g/L，4月18日至4月20日共输注红细胞11 U。4月21日患者大腿伤口处无活动性出血，坏死物质较多，APTT 60.6 s，D-二聚体5 980 µg/L，HGB升至90 g/L，继续加强换药，PCC减量至3 300 U，血培养提示肺炎克雷伯杆菌，4月22日患者无发热，APTT 52.1 s，D-二聚体7 779 µg/L，凝血酶原复合物减量至3 000 U。4月25日APTT 49.1 s，D-二聚体6 792 µg/L，PCC减量至1 800 U，下肢术区无活动性出血，FⅧ∶C 75.4％，FⅧ抑制物0 Bethesda，暂停输注PCC，甲泼尼龙减量至32 mg/d。4月27日APTT降至正常范围。1周后复查FⅧ抑制物仍为0 Bethesda，停用环磷酰胺，甲泼尼龙每周减量8 mg至停服，每周复查下肢静脉超声无血栓形成。4月28日由重症医学科转入烧伤科病房。5月6日在神经阻滞+静脉麻醉下行头部取皮+右大腿创面清创、右侧股四头肌修补、局部皮瓣转移、自体游离皮片移植+负压装置置入术，术后给予补液、右下肢创面负压吸引、维护多脏器功能等治疗。5月13日在静脉麻醉下行右大腿慢性创面清创、右侧股四头肌修补、局部皮瓣转移+负压装置置入术，5月22日在全麻下行头部取皮+右大腿慢性创面清创自体游离皮片移植+负压装置置入术，5月29日在全麻下行右大腿慢性创面清创坏死组织去除+负压装置置入术，6月5日拆除负压装置，皮肤创面愈合，拆除负压装置后3周，皮肤全部愈合，植皮全部成活。

## 讨论及文献复习

AHA是因循环血中出现抗FⅧ自身抗体导致FⅧ∶C降低的获得性出血性疾病。AHA的发病率较低，每百万人中1.5例[1]。AHA最常见于老年人（中位年龄64～78岁），也可见于儿童和围产期患者[2]。实验室表现为孤立性APTT延长，大多数患者表现为严重的自发性或创伤后出血，但有些患者可能无症状[3]。由于AHA具有罕见、突发及出血异质性大的特点，并且有时患者首诊并非在血液科而导致诊断延迟，因此国内外对于该病的认识均有待提高[4]。诊断延迟的患者有较高的死亡率[5]。本例患者为老年男性，无基础疾病，既往有手术史，但围手术期无异常出血，入院当时APTT正常，住院过程中监测APTT延长，并且有出血表现，且有创操作后出血不止，FⅧ∶C下降，FⅧ抑制物阳性，明确诊断AHA。CARE研究显示患者首次出血至确诊的中位时间为30 d，就诊时严重出血的患者占60.9％[6]，本例患者自出血至明确诊断时间为15 d。

临床中遇到以下情况时需要考虑AHA的诊断：①既往无出血史的非血友病患者（尤其是老年人或者产后）出现自发性出血或外伤、有创操作后发生与预期不符的过度出血，合并不能解释的孤立性APTT延长；②术前发现不能解释的孤立性APTT延长[7]。患者在外院发现APTT延长，当时考虑是否存在头孢哌酮/舒巴坦影响凝血功能，关于头孢哌酮/舒巴坦影响APTT的可能机制主要为头孢哌酮的第3位侧链上N-甲硫四氮唑基团，该基团在肝脏微粒体竞争性结合谷氨酸γ梭化酶，导致维生素K依赖性凝血因子生成障碍。该基团可抑制维生素K氧化还原酶，阻断维生素K循环，导致参与凝血因子活化的还原型维生素K缺乏[8]。另外。头孢哌酮主要经胆道排泄，易使肠道正常菌群受抑，影响凝血因子的合成从而引起凝血功能异常。

AHA的病例中约有50％为特发性，未发现潜在原因[9]，大约有50％的AHA患者可以发现病因或基础疾病，欧洲获得性血友病登记研究（EACH2）的结果表明，与AHA相关的可能疾病中48.1％（125/260）为自身免疫病和恶性肿瘤，自身免疫病包括系统性红斑狼疮、类风湿性关节炎、银屑病、天疱疮等；肿瘤性疾病包括血液肿瘤如慢性淋巴细胞性白血病、非霍奇金淋巴瘤、多发性骨髓瘤[10]，在实体瘤中，前列腺癌、肺癌和结肠癌较多见[11]。感染也是AHA相关诱发因素，包括急性乙型及丙型肝炎病毒感染、HIV、流感，呼吸系统疾病如支气管哮喘、慢性阻塞性肺疾病[12]。本例患者无自身免疫病及肿瘤性疾病基础，除入院后肺炎外无其他感染性疾病病史。药物是诱发AHA的重要因素之一，药物相关的AHA（D-AHA）在目前文献报道的药物包括抗生素，如青霉素、磺胺类药物，苯妥英、氯霉素、甲基多巴、干扰素α、氟达拉滨等[13]。一项研究中，在WHO全球药品潜在效应报告数据库（VigiBase）收集了2004年7月至2021年11月共185例与AHA显著相关的药物病例表明，D-AHA男性发生率（59％）高于女性（41％），中位年龄75（8～98）岁，中位时间从疑似药物开始为30（9.5～73.75）d，数据库中报道更新了药物，包括阿仑妥珠单抗、氯吡格雷、奥马利珠单抗、乙酰水杨酸酸、聚乙烯干扰素、阿尔法、华法林、阿哌沙班、阿莫西林、西格列汀、利巴韦林、尼伏鲁单抗、新冠肺炎疫苗、哌拉西林他唑巴坦、环丙沙星，其中氯吡格雷、阿伦单抗、奥马珠单抗是首次提出D-AHA显著药物警戒信号的药物[14]。本例患者先后应用哌拉西林/他唑巴坦、头孢哌酮/舒巴坦、去甲万古霉素、亚胺培南、利奈唑胺、莫西沙星、左氧氟沙星等，不能除外药物所致凝血异常。另外手术或创伤可能是AHA致病因素。目前文献均为病例报道，Hidalgo等[15]报道1例79岁老年男性摔伤后出现腹膜后血肿，出血量极大，FⅧ∶C 2％，FⅧ抑制物24.4 BU/ml，确诊为AHA，应用糖皮质激素及环磷酰胺后出血减少，但患者出院后几周于家中去世。手术是AHA的促发因素，可能与各种病理性，外伤性和手术性的组织损伤诱发有关，这些组织损伤有可能激活机体的凝血途径，活化的凝血因子因结构的改变，使其抗原性增强[16]。综合分析，本例患者创伤、手术、感染、药物因素均有可能是AHA的致病因素。

AHA的管理有两个主要目标：旁路止血及去除抑制物。出现严重出血并发症的AHA患者应使用旁路剂进行治疗，如活化凝血酶原复合物（aPCC）或rFⅦa[17]，这两种药物都已成功使用了数十年，并且止血效果良好，aPCC可以每天给予2～3次，剂量为50～100 U/kg，每天最大量不超过150 U/kg。rFⅦa 90 µg/kg，每2～3 h 1次静脉注射直至止血。aPCC和rFⅦa给药持续时间取决于出血的类型和严重程度，在临床中2种药物可以交替使用，应用PCC和rFⅦa主要不良反应为血栓形成，rFⅦa在多数研究中的止血有效率>90％，血栓栓塞事件发生率为0～2.9％[18]–[19]。CARE研究中，PCC单药止血有效率为84.6％，中位止血时间为72 h，血栓事件发生率为8.8％[6]。在使用PCC过程中应注意监测血栓事件的发生。根据PCC产品中FⅦ含量的差异，PCC可分为三因子（FⅦ含量低）和四因子（FⅦ含量高）产品，目前尚无两种类型PCC疗效差异的研究数据。国内尚无aPCC产品供应，如由于药物可及性或经济原因无法持续应用rFⅦa，可以考虑与PCC序贯使用[4]。本例患者为老年男性，卧床，下肢骨折术后，下肢肌间静脉血栓，无深静脉血栓，无基础疾病，为血栓高风险患者，因经济因素，首次给予rFⅦa 5 mg静脉注射，未予足剂量应用，序贯PCC旁路止血，按30 U/kg替代治疗后仍有出血，加量至3600 U，期间监测D-二聚体较基线无显著升高，未发生血栓事件。艾美赛珠单抗是一种双特异性FⅧ模拟物，在血友病患者中，无论是否伴有抑制物均可使用，但不包括AHA。Knoebl等[20]用艾美赛珠单抗成功治疗12例AHA患者，皮下注射3 mg/kg每周1次，共2～3次，随后1.5 mg/kg每3周1次，可使APTT恢复正常并止血。艾美赛珠单抗价格昂贵，目前临床上应用较少。

所有患者在确诊后首先应去除诱因及治疗基础疾病，此外应立即采用免疫抑制疗法（IST）清除FⅧ抑制物。一线治疗方案包括糖皮质激素单药、糖皮质激素联合环磷酰胺、糖皮质激素联合利妥昔单抗，中位部分缓解时间分别为56、42、42 d。本例患者糖皮质激素联合环磷酰胺治疗1周后抑制物转阴，起效较快，可能的原因：①患者可能为多种因素所致AHA，但入院后诱因逐渐去除；②部分AHA可能为自限性；③可能与患者治疗前抑制物滴度较低有关。本例患者在抑制物转阴后糖皮质激素每周减量，4周后减停，此后监测FⅧ∶C均正常，抑制物持续阴性，疾病达CR。

AHA是一种罕见的疾病，容易发生诊断延迟，死亡率较高。对于出现严重出血症状的患者应高度警惕AHA，尤其孤立性APTT延长的患者。AHA治疗成功的关键在于及时诊断、及早给予恰当的治疗，在实施有效的替代治疗前禁行有创操作，当合并外伤需要处理时需要多学科协作，血液科医生需全程参与，制定规范的替代治疗方案。
